# From the Editor‐in‐Chief

**DOI:** 10.1111/pbi.14273

**Published:** 2023-12-28

**Authors:** Johnathan Napier

2023 is the first year that I had the privilege of serving as Editor‐in‐Chief for Plant Biotechnology Journal and in this brief reflection on the last 12 months, I want to firstly pay tribute to Professor Henry Daniell, our preceding EiC who successfully guided PBJ to the esteemed position the journal currently holds. We all know that these achievements do not happen by magic, so it is a testament to Henry's commitment, along with the expert and unstinting contributions of the Senior Editors and Associate Editors that compromise our Editorial Board, that PBJ is now firmly established as a leading journal in the Plant Sciences. So, a heartfelt ‘thank you’ to the Editorial Board. Equally, the peer‐review process is completely dependent on reviewers providing their time and knowledge pro bono, and while we all know that myriad demands on our time seem to continually increase, it is great that we still can obtain high‐quality and useful feedback on submissions. So, an additional ‘thank you’ to all our reviewers.

My ambition for PBJ is to maintain our reputation as a top‐quality journal, but also to use the journal to better support our research community. I would like to see PBJ as the cornerstone of the plant biotechnology community, helping to advance our science but also mentoring and advising the next generation of researchers. Some of the Editorial Board met recently in London and we discussed ideas for how we might achieve this, so hopefully 2024 will see some exciting changes and additions.

All the Best for the coming year,

Johnathan

